# Fermented Rapeseed Meal as a Component of the Mink Diet (*Neovison vison*) Modulating the Gastrointestinal Tract Microbiota

**DOI:** 10.3390/ani11051337

**Published:** 2021-05-08

**Authors:** Łukasz Wlazło, Bożena Nowakowicz-Dębek, Anna Czech, Anna Chmielowiec-Korzeniowska, Mateusz Ossowski, Marek Kułażyński, Marcin Łukaszewicz, Anna Krasowska

**Affiliations:** 1 Department of Animal Hygiene and Environmental Hazards, University of Life Sciences in Lublin, Akademicka 13, 20-950 Lublin, Poland; lukasz.wlazlo@up.lublin.pl (Ł.W.); anna.korzeniowska@up.lublin.pl (A.C.-K.); 2 Department of Biochemistry and Toxicology, University of Life Sciences in Lublin, Akademicka 13, 20-950 Lublin, Poland; anna.czech@up.lublin.pl; 3 Department of Fuel Chemistry and Technology, Faculty of Chemistry, Wrocław University of Science and Technology, Gdańska 7/9, 50-344 Wrocław, Poland; marek.kulazynski@pwr.edu.pl; 4 InventionBio, Wojska Polskiego 65, 85-825 Bydgoszcz, Poland; marcin.lukaszewicz@uwr.edu.pl (M.Ł.); anna.krasowska@uwr.edu.pl (A.K.); 5 Department of Biotransformation, Faculty of Biotechnology, University of Wroclaw, F. Joliot-Curie 14A, 50-383 Wrocław, Poland

**Keywords:** *Neovison vison*, fermented rapeseed meal, GIT microbiota, GMO alternatives

## Abstract

**Simple Summary:**

The high protein requirement in the diet of mink is currently met using extruded cereals, meat and bone meal, which raises the cost of mink farming. At the same time, there are waste products that may contain many infectious agents. Therefore, there is a need to look for high-protein feeds with properties stabilizing the gastrointestinal tract (GIT). Fermented rapeseed meal (FRSM) may prove to be such a material. During the fermentation process, rapeseed loses the anti-nutritional substances contained in it, while acquiring new properties of a bioproduct. A study was carried out in mink to determine how FRSM in the diet modifies the population of microorganisms inhabiting the GIT and stabilizes its microbiota. FRSM was included in the feed of experimental animals, and changes in the population size of groups of intestinal microorganisms were analysed in relation to the control group receiving standard feed. Microbiological analyses were performed on faeces samples and from intestinal contents collected after the animals had been euthanized. The paper presents the effect of supplementation with FRSM obtained using biotechnological methods and demonstrates that it can be used in animal diets as a bioproduct influencing the gastrointestinal microbiota.

**Abstract:**

Fermented rapeseed meal (FRSM) was used in the diet of American mink (*Neovison vison*). An advantage of this product is its prebiotic and functional properties, which can modify the bacterial microbiota of the GIT. A control group and three experimental groups were formed, with 60 animals in each group. The control group received a basal diet and the experimental groups received a diet with a 2%, 4% or 6% of FRSM as a replacement of extruded wheat. *Bacillus subtilis* strain 87Y was used to ferment the rapeseed meal (RSM). The study was conducted on mink from the age of 16–17 weeks until slaughter. Changes in the microbiota were analysed in samples of the animals’ faeces and intestinal contents. The analyses included determination of the total number of bacteria and fungi, the number of coliforms and *Escherichia coli*, the total number of anaerobic *Clostridium perfringens*, and the presence of *Salmonella* spp. In animals receiving 4% and 6% FRSM (groups II and III), the content of microscopic fungi and the number of *C. perfringens* bacteria was significantly (*p* ≤ 0.05) lower than in the animals from the control group (group 0). A decrease in *E. coli* was observed in all experimental groups (I, II and III), although these differences were not statistically significant. The inclusion of FRSM in the feed ration did not affect the number of lactic acid intestinal bacteria. Analysis of the results obtained from the stool samples showed that the inclusion of FRSM in the ration did not significantly affect the number of microorganisms in each group. However, as in the case of the intestinal contents, in these samples there was a decrease in the total number of *C. perfringens* in the experimental groups (I, II and III), with a simultaneous increase in the number of mesophilic bacteria in relation to the control. There was no detection of *Salmonella* bacteria in any of the analysed material.

## 1. Introduction

The dynamic growth of animal production, especially in rapidly developing countries, has increased demand for high-quality, protein-rich fodder, which may include rapeseed meal (RSM). Unfortunately, its use is significantly limited by its high fibre content and the presence of anti-nutritional compounds, including glucosinolates, tannins, phytates, and many others [1]. A new approach which solves the problem of the limited use of RSM is its fermentation [2,3]. In the fermentation process rapeseed protein is hydrolysed by microorganisms, and anti-nutritional substances are inactivated and eliminated. Microorganisms, as a source of enzymes such as glucosidase, amylase, cellulase, chitinase, phytase, xylanase, esterase, invertase or lipase, can effectively detoxify the meal and increase its biological value. An additional advantage of microbial fermentation of RSM is that it provides a product with functional properties associated mainly with the production of beneficial (antipathogenic) bacteriocins and probiotic properties supporting digestion and modifying the natural gastrointestinal tract (GIT) microbiota, which has been confirmed in poultry and pigs [4,5]. Despite numerous strategies to control and prevent microbial contamination, GIT bacterial infections remain a challenge for the feed industry. The composition and functioning of the GIT microbiota are crucially influenced by diet. Therefore, livestock farmers often use feed supplements such as probiotics, prebiotics or organic acids to prevent GIT bacterial infections and improve GIT function and the microbial ecosystem in ruminants and monogastric animals [6,7]. Fermented rapeseed meal (FRSM) is a bioproduct which due to its high content of lactic acid can act as an effective stimulator of the GIT microbiota. Probiotic strains of microorganisms, i.e., *Lactobacillus fermentum* and *Bacillus subtilis*, can be used in the fermentation process. Studies confirm that they reduce pathogenic bacteria of the GIT, including *Salmonella* spp. and *Escherichia coli*, and are involved in the production of bioactive rapeseed peptides that improve immune function [8]. High content of lactic acid inhibits the growth of fungi, which has been exploited in various cereal-based fermented products [9]. Researchers have identified a large number of strains of lactic acid bacteria with a broad spectrum of antifungal activity and interaction with mycotoxins, leading them to be inactivated or removed by binding to cell walls [10]. Numerous studies indicate that fermented feed components exert a beneficial effect on the health and productivity of animals. In a study by Bai et al. [11], the use of FRSM in poultry feed increased productivity and stimulated immune function and antioxidant capacity in the birds. A study on the use of an FRSM supplement in fish showed significantly increased bactericidal activity of lysozyme and peroxidase and high resistance to oxidative stress in specimens for which fish meal was replaced in the amount of 25% and 50% with FRSM [12]. A beneficial effect of fermented feed components on the composition of the GIT microbiota has been shown in both poultry and pigs, with a reduction in the number of coliforms and *Salmonella* spp. [13,14]. There is no research on the use of FRSM in the diet of carnivorous fur-bearing animals, especially mink [15,16].

The GIT microbial community composition of carnivores in general appears distinct from that of omnivores and herbivores [17,18]. In the specific case of the microbiota of mink (*Neovison vison*), culturing has shown the highest bacterial load in the colon, with up to 10^8^ CFU/g, which is approximately 2–4 orders of magnitude lower than in many other mammals, possibly due to the short intestinal tract of mink and rapid transit in the GIT [19]. A recent culture-based study of the anaerobic and microaerophilic bacteria in faecal samples from ferrets (*Mustela putorius furo*) found that *Clostridium acetobutylicum* and *Helicobacter* spp. were isolated most frequently [20]. In carnivores, lactobacilli are never common isolates, but are detected more often after weaning. Due to the specific character of the diet of carnivores, total bacterial densities and the relative proportions of groups of intestinal bacteria differ from those known for other mammals [21]. The health-promoting effect of fermented diet components is often attributed to high concentrations of lactic acid bacteria. These bacteria, by colonizing the GIT, impede adhesion of pathogens to the intestinal epithelium, and by acidifying the environment give rise to unfavourable conditions for the development of pathogenic microbiota [22]. Previous studies on the effect of FRSM on the GIT microbiota have often focused only on reducing pathogenic bacteria such as *E. coli* and *Salmonella* spp., and most of them have been conducted in poultry, distinguished by a specific type of digestive system. Therefore, the aim of the study was to assess the effect of the inclusion of FRSM in feed on the population size of microorganisms colonizing the GIT of mink.

## 2. Materials and Methods

### 2.1. Preparation of Fermented Rapeseed Meal (FRSM)

*B. subtilis* strain 87Y from the strain collection of Invention Bio Ltd. (Bydgoszcz, Poland) was used for the fermentation of RSM [23]. A preculture was grown on Lysogeny Broth (LB) agar (10 g/L NaCl, 10 g/L peptone, 5 g/L yeast extract), and then a bacterial suspension with OD_600_ = 0.1 was prepared in MIM1 medium (8.4 g/L Na_2_HPO_4_, 3.9 g/L NaH_2_PO_4_, 2.3 g/L urea, 0.5 g/L MgSO_4_, 60 g/L sucrose, 1.2 mg/L FeSO_4_, 1.6 mg/L CuSO_4_, 5 mg/L MnSO_4_). The RSM was pasteurized for 8 min at 80 °C, transferred to a bioreactor, and inoculated with a previously prepared bacterial suspension in a 1:1 ratio under 50% moisture. Fermentation was carried out for 24 h at 37 °C with continuous shaking (20 rpm). Moisture was maintained at 50% by adding sterile water and with 50 L/min aeration. Following the fermentation process, the RSM was dried using a fluid bed drier to a moisture level of 10–11%. Wet FRSM contained 2.25 × 10^8^ CFU of *B. subtilis*, which dropped to 5 × 10^7^ CFU after the drying process. The chemical composition of RSM before and after fermentation is shown in Table 1. Dry matter, crude ash, crude protein, ether extract, and crude fibre were determined according to Latimer [24] and the lactic acid level according to Taylor [25].

### 2.2. Experimental Animals and Diet

The experiment was carried out on mink (*Neovison vison*) of the pastel variety on a farm located in south-eastern Poland. The microclimatic conditions were typical for that region during the autumn and winter. During this period, the temperature ranges from 0 to 10 °C, humidity is about 85%, and air movement is about 3.5 m/s. The animals were housed in cages in a shed system with ad libitum access to water. In accordance with guidelines regarding the welfare of fur-bearing animals, the cages were 45 cm high, 30 cm wide and 90 cm long. Each cage was connected to a wooden nesting box 20 cm high, 28 cm wide and 23 cm long. All cages were equipped with a shelf on which the animals rested. Throughout the study period, they were under veterinary care with prophylaxis appropriate for the species. At 6–7 weeks of age, all animals were dewormed and vaccinated against botulism, viral haemorrhagic enteritis, and haemorrhagic pneumonia. At 9–10 weeks of age, the animals were vaccinated against canine distemper and dewormed a second time. Once every three months, veterinary inspection employees conducted an assessment of hygiene and sanitation on the farm. In the event of diseases, clinical examinations of animals and necropsies of dead animals were performed in order to select a treatment method. During the study period, no cases requiring treatment or administration of antibiotics were observed in the experimental animals. The study was conducted on 4 groups of animals, with 60 animals in each group and equal numbers of each sex (30 males and 30 females). The mink were randomly assigned to the research groups. There were 6 replications of 10 individuals in each group. The control group (group 0) comprised mink that received a basal diet with no additives (Table 2).

The mink in the experimental groups received feed with a 2% (group I), 4% (group II), or 6% (group III) share of FRSM. FRSM was introduced in place of extruded wheat and the chemical composition of the feed ration in all groups was balanced according to mink nutrition standards for each feeding period [26]. All raw ingredients were ground, mixed and homogenized with the addition of vitamins and preservatives, and then given to the animals in wet form on the cages. The chemical composition of the feed ration was balanced by adding an appropriate amount of meat and bone meal (for protein) and extruded wheat (for carbohydrates), as shown in Table 3.

All tests on animals were conducted with the consent of the Local Ethics Committee on animal experiments (approval no. 50/2018, of 1 April 2018). The animals were fed ad libitum by placing 250 g of feed per animal on the top of the cages, so that the animals had access to it throughout the day. The experiment was carried out on young mink aged 16–17 weeks from 1 September to the beginning of December, when the mink were slaughtered as part of the technological process.

### 2.3. Sampling for Analysis

Prior to the start of the feeding experiment, a microbiological analysis of the FRSM was performed to determine its hygienic condition and the concentration of probiotic microorganisms. During the experiment, at two-week intervals, faeces samples were collected by suspending foil under the cages so that the animals’ faeces dropped onto it. In the morning faeces were collected into prepared containers and immediately subjected to microbiological analysis. Faecal samples were collected for microbiological analyses three times from under the cages of all animals from each group. Thirty individual faeces samples were collected from each group of animals into disposable sterile containers. The samples were cooled to 4–6 °C and transported in a thermal bag to the laboratory, where they were homogenized into one aggregate sample for each group for the microbiological analyses. Immediately after slaughter, the animals were eviscerated, and the contents of the GIT (jejunum, ileum and colon) of 6 animals from each group were collected into sterile containers. The material was immediately placed in thermal bags and transported to the laboratory, where it was analysed within about two hours after slaughter. A full microbiological analysis of the samples (faeces and digestive contents) was carried out in accordance with national recommendations and applicable standards for each group of microorganisms [27,28,29,30].

### 2.4. Microbiological Procedures

The mink faeces and the intestinal contents were pooled and 20 g samples of each type of material were weighed out and placed in sterile bottles containing 180 mL of Ringer’s solution. The solution was shaken for 5 min and allowed to settle for 15 min. Then, a series of tenfold dilutions of the test samples was prepared in physiological saline solution and plated on previously prepared Petri dishes with an appropriate microbiological medium.

1. The total number of mesophilic aerobic bacteria, on enriched agar medium for 48 h at 37 °C (BTL Ltd., Łódź, Poland);
2. The total number of fungi, on Sabouraud agar, for 5–7 d at 25 °C (BTL Ltd., Łódź, Poland);
3. The total number of coliform bacteria on Endo LES agar, for 24 h at 37 °C (BTL Ltd., Łódź, Poland);
4. The total number of *E. coli*, on mFC agar for 18–24 h at 44 °C (BTL Ltd., Łódź, Poland);
5. The total number of *C. perfringens*–sulphate-reducing bacteria growing in anaerobic conditions, on iron sulphite agar (TSC) for 48 h at 37 °C (BioMerieux, Marcy l’Etoile, France);
6. The total number of lactic acid bacteria of the genus *Lactobacillus*, on MRS agar for 3–5 d at 30 °C (BTL Ltd., Łódź, Poland);
7. The presence of *Salmonella*, on SS agar (*Salmonella*-*Shigella* and XLD) after prior multiplication of samples in buffered peptone water and Rappaport-Vassiliadis broth (BTL Ltd., Łódź, Poland) for 24 h at 37 °C. Final identification was carried out using API tests (BioMerieux, Marcy l’Etoile, France) and polyvalent sera (Biomed Inc., Kraków, Poland).

Each microbiological assay was performed by plating in duplicate for each dilution. After a specified incubation time, the colonies were counted using a Scan 300 automatic counter (Interscience, Saint Nom la Brétèche, France) and converted to 1 g of test material according to ISO 4832 [31] and ISO 7218 [32]. Results are expressed as colony forming units per g [CFU/g] and presented in the tables.

### 2.5. Statistical Analysis

The obtained research results were analysed statistically. The normality of the distribution was assessed by the Shapiro–Wilk test. If the distribution was normal, one-way analysis of variance (ANOVA) was performed. Fisher’s F-test was used to analyse the homogeneity of variance. When a significant treatment effect was noted, the post hoc Tukey test was used to determine differences between groups. The calculations were made with the statistics package Statistica v. 9.1. (StatSoft Inc., Tulsa, OK, USA). Data were presented as means and standard error of the mean (SEM). Values were considered to differ significantly at *p* ≤ 0.05. Values designated with a different superscript letters a-c within a row differ significantly (*p* ≤ 0.05).

## 3. Results

### 3.1. Effect of Fermentation on the Chemical Composition and Microbiological Quality of Rapeseed Meal (RSM)

The effect of fermentation on the chemical composition of RSM is presented in Table 1. The fermentation process slightly increased protein and ash content and reduced fat, fibre and dry matter content. The increase in the most important components, protein and lactic acid, can be explained by the growth of bacteria during the fermentation process. Microbial synthesis of proteins together with the biomass of microorganisms, which consist primarily of proteins, increased the share of these components. No mesophilic aerobic bacteria or fungi were found in the tested batch of FRSM. The material was free of contamination, i.e., coliform bacteria, *E. coli*, *C. perfringens* and *Salmonella*. The total number of lactic acid bacteria of the genus *Lactobacillus* was determined to be 2.2 × 10^5^ CFU/g of product.

### 3.2. Populations of Intestinal Microorganisms in the Experimental Animals

The results of the microbiological tests of the mink intestinal contents and faeces are presented in Table 4 and Table 5. The results indicate that a 6% share of FRSM in the feed (group III) reduced the total count of mesophilic bacteria in the intestinal contents, but the differences were not statistically significant (*p* ≤ 0.05), see Table 4. In animals receiving 4% and 6% FRSM (groups II and III), the content of microscopic fungi was significantly (*p* ≤ 0.05) lower than in the animals from the control group (group 0). The statistical analysis also showed a significantly (*p* ≤ 0.05) higher number of coliforms in the control group than in experimental group II (4% FRMS).

A decrease in *E. coli* was observed in all experimental groups (I, II and III), although these differences were not statistically significant (*p* ≤ 0.05). The inclusion of FRSM in the feed ration did not affect the number of lactic acid intestinal bacteria (Table 4). A decrease in the number of *C. perfringens* bacteria was noted in the intestinal contents of animals receiving feed with FSRM. The statistical analysis showed statistically significantly lower counts of *C. perfringens* bacteria in groups II (4% FRMS) and III (6% FRMS) than in the control group (*p* ≤ 0.05), see Table 4.

### 3.3. Numbers of Microorganisms in the Faeces of the Experimental Animals

The ANOVA statistical analysis of the results obtained from faecal samples showed that the inclusion of FRSM in the feed ration did not significantly (*p* ≤ 0.05) affect the number microorganisms in each group (Table 5). Fewer mesophilic bacteria were isolated from the faeces of group III mink than from faeces from the group of experimental animals receiving feed with a 4% share of FRSM (group II), but the differences were not statistically significant (*p* ≤ 0.05). In the samples analysed, as in the case of the intestinal contents, there was a decrease in the number of *C. perfringens* and an increase in the number of mesophilic bacteria in the experimental groups (I, II and III) relative to the control. In this case as well, the differences were not statistically significant at *p* ≤ 0.05 (Table 5). In the case of the other analysed parameters, the addition of FRSM to the feed was not shown to influence the numbers of microorganisms in the faeces. The differences did not exceed the threshold for statistical significance. 

There was no detection of *Salmonella* bacteria in any of the analysed material.

## 4. Discussion

Due to the short digestive tract of carnivores and the rapid rate of food passage in the intestines, the microbiological composition of the GIT is largely determined by the microorganisms ingested with the feed [19,21]. This suggests the need to introduce beneficial microbiota to inhabit the intestines of animals together with their feed. There are increasing reports on the possibility of including FRSM in animal diets [5,22,33,34]. The positive effect on the GIT microbiota of animals receiving FRSM is often attributed to the increase in the population of lactic acid bacteria [14]. In our study, there were no significant differences in the numbers of these microorganisms between the groups receiving and not receiving FRSM, but a much higher proportion of lactic acid formed in the fermentation process was observed in FRSM. Williams et al. [19] show a low level of lactic acid bacteria in the digestive tract of mink. The authors found that lactobacilli were never common isolates, but were detected more often after weaning, particularly in adults fed diets containing additional fibre. These data are in conflict with research results [20] indicating that after *Clostridium*, lactic acid bacteria were the most commonly isolated anaerobic bacteria in ferrets. Research by Rajilić-Stojanović and de Vos [35] based on 16S rRNA gene sequencing found very low relative numbers of lactic acid bacteria and no bifidobacteria. The reasons for this discrepancy may include a relatively high level of detection of the sequencing approach compared to traditional microbial cultures. The microorganisms used in the fermentation process alter the pH by acidifying the environment. According to Niba et al. [36], a reduction in pH to below 4.5 inhibits the growth of pathogenic microbiota. Acidification of the upper GIT creates favourable conditions for maintaining the balance of the microbiota and increases the amount of lactic acid, which is believed to reduce bacteria of the family *Enterobacteriaceae* [22]. An increase in the amount of lactic acid creates an unfavourable environment for the development of fungi [37]. This was observed in the present study as well, as the number of fungi decreased as the share of the fermented component in the diet was increased. This phenomenon is linked to the interactions between microbial populations. The numerous probiotic bacteria on the intestinal epithelium create a competitive niche, which according to the principle of exclusion creates a defensive barrier against infection and pathogenic bacteria such as *Salmonella* and *E. coli* [33,38,39].

The microbiological analyses did not show the presence of *Salmonella* in any samples of either faeces or intestinal contents taken from the animals. Research conducted on poultry by Heres et al. [40] indicates that fermented feeds may reduce *Salmonella enteritidis* in poultry flocks and the number of *Salmonella* in the caecum contents of animals. Acidification of the GIT of broiler chickens by organic acids has been shown to improve its resistance to infection even after oral administration of *Salmonella* [22,41]. An important epizootic problem associated with animal feeding is anaerobic infections. A condition for the growth of all anaerobic microorganisms in tissues is their hypoxia and decreased redox potential (Eh). In healthy tissues, the redox potential ranges from 126 to 248 mV, while in anaerobic conditions Eh is close to zero or negative (even up to −50 mV in tissues of dead animals) [42]. Mink feed consisting largely of by-products of animal slaughter, together with the digestive tract contents, creates favourable conditions for the development of pathogenic anaerobic microbiota. *C. perfringens*, which produces a total of 15 toxins in various combinations, including the lethal toxins perfringolysin O (PFO), enterotoxin (CPE) and beta2 toxin (CPB2), can be considered the most important and most common species of the genus *Clostridium*. These toxins are often produced in the GIT as a result of a change in diet or other environmental factors [42,43]. We found no studies in the available literature on the effects of fermented protein feed components on the number of anaerobic bacteria in animals. A number of specific antimicrobial properties are ascribed to fermented products owing to the antimicrobial metabolites produced. Numerous organic acids and bacteriocins are used to combat infections with *Clostridium* ssp. Research suggests the prospect of expanding the use of beneficial lactic acid bacteria (LAB) and their metabolites to include potential application in unconventional treatment and prevention of inflammatory and infectious intestinal diseases [44]. In the present study, a reduction in the number of *C. perfringens* anaerobic bacteria was observed in all experimental groups. Frequent anaerobic microbiota colonization in the intestines of carnivores is reported by Gugołek et al. [18] and Handl et al. [45], who indicate that microorganisms from the Clostridia class of Firmicutes are associated with the GIT mucosa. Recent research on anaerobic and microaerophilic bacteria in ferret (*Mustela putorius furo*) faeces showed *C. acetobutylicum* and *Helicobacter* spp. to be the most common isolates [20]. Bahl et al. [21] report a significant share of anaerobic bacteria permanently residing in the mink digestive tract. Analysing the effect of feed type and fasting on the composition of the intestinal microbiota, the authors found that Clostridia, Gammaproteobacteria and Fusobacteria predominate in the average bacterial composition of the intestines both after fasting and after feed consumption. Although anaerobic bacteria are frequently indicated as potentially pathogenic microorganisms, their pathogenicity in mink requires further research. However, a large increase in *C. perfringens* within the microbiota or intestinal epithelium may alter intestinal permeability, leading to chronic degenerative processes. Due to chronic inflammation and disturbance of the intestinal microbiota, villus height decreases, cell turnover increases, and digestive and absorption capacity is reduced, resulting in a decrease in the animals’ productivity [46].

Many researchers are currently placing particular emphasis on the potential of antimicrobial metabolites produced by certain LAB strains and their use as active nutritional and therapeutic agents. The search for suitable, safe and nutritionally beneficial feed components enables their potential application as ‘biotherapeutics’ in both human and veterinary medicine. The intestinal microbiome is currently regarded as a promising target for future unconventional treatment of inflammatory and infectious diseases in livestock. As new innovative applications of fermented products in feeding practice emerge, animal breeders seek feed components that can be used as alternative therapeutic agents (in contrast to antibiotics and chemical drugs). A challenge for the natural sciences is thus to identify probiotic microbes which, in addition to producing organic acids, will exhibit antimicrobial activity against pathogenic microbes. This shift in the interest of researchers and the accumulation of knowledge of the applications of fermented feed components will enable changes in longstanding agricultural practice. To achieve the intended effect, however, there is a need for interdisciplinary studies to assess the safety of these products at the intraspecies level prior to their used in farm production conditions.

Combining knowledge on animal production and the use of fermented feed components and their properties seems to be a promising and innovative approach which may also have potential application in synergistic combination with antibiotics in the treatment of intestinal dysbiosis.

To the best of our knowledge, this is the first study to use FRSM in the diet of mink to characterize the GIT microbiota. The study included only a small number of mink, which were apparently healthy and showed high inter-individual variability. Nevertheless, the differences observed in the GIT microbiota between groups are interesting and may contribute to new prophylactic and therapeutic solutions in the breeding of carnivorous fur animals.

## Figures and Tables

**Table 1:** The analysed nutritive value of raw or fermented rapeseed meals (g kg^−1^ of diet).

Parameter	RSM	FRSM
Dry matter	883.5	868.7
Crude ash	70.7	73.15
Crude protein	337.50	345.95
Crude fibre	143.75	141.55
Ether extract	24.05	16.3
Lactic acid, g kg^−1^	0.62	3.83
Nitrogen-free extract	307.5	291.75

RSM—rapeseed meal; FRSM—fermented rapeseed meal.

**Table 2:** Composition of the feed ration for mink during the experiment (% share).

Ingredient	% Share of Feed			
	Group 0	Group I	Group II	Group III
Turkey bones	28	28	28	28
Poultry intestines	55	55	55	55
Soybean oil	1	1	1	1
Extruded wheat	13	11	9	7
Haemoglobin powder	1	1	1	1
Meat and bone meal	1	1	1	1
Sugar beet pulp	1	1	1	1
FRSM	0	2	4	6

FRSM—fermented rapeseed meal.

**Table 3:** Mean content of nutrients in 1 kg of mink feed (g).

Ingredient	Group 0	Group I	Group II	Group III
Dry matter	438.8	432.7	418.7	414.1
Crude ash	48.9	46.6	46.,7	45.2
Crude protein	157.6	164.7	153.8	151.5
Crude fibre	19.8	22.7	27.1	23.9
Ether extract	134.7	130.9	106.0	144.9
Dry matter	438.8	432.7	418.7	414.1

**Table 4:** The number of microorganisms in individual groups in the intestinal content of the animals (CFU/g).

Parameter	Experimental Group (n6)				SEM	*p*-Value
	0	I	II	III		
Total number of mesophilic bacteria	1.8 × 10^6^	1.4 × 10^6^	9.0 × 10^5^	7.6 × 10^5^	1.1 × 10^5^	0.548
Total number of fungi	7.7 × 10^2 a^	9.1 × 10^2 a^	2.0 × 10^2 b^	1.1 × 10^2 c^	4.2 × 10^1^	0.005
Total number of coliforms	7.0 × 10^5 a^	1.0 × 10^6 a^	2.9 × 10^5 b^	5.7 × 10^5^	4.1 × 10^4^	0.050
Total number of *E. coli*	4.8 × 10^5^	2.7 × 10^5^	3.8 × 10^5^	1.3 × 10^5^	4.9 × 10^4^	0.121
Total number of lactic acid bacteria of the genus *Lactobacillus*	9.3 × 10^5^	7.0 × 10^5^	5.7 × 10^5^	4.3 × 10^5^	3.7 × 10^4^	0.187
Total number of *C.* *perfringens*	3.1 × 10^4 a^	3.9 × 10^4 a^	1.2 × 10^4 b^	1.1 × 10^4 c^	1.5 × 10^3^	0.006

SEM–Standard error of the mean. ^a–c^–Values within a row with different superscripts differ significantly (*p* ≤ 0.05). Groups: 0—control; I—group receiving a diet with 2% FRSM; II—group receiving a diet with 4% FRSM; III—group receiving a diet with 6% FRSM.

**Table 5:** Numbers of microorganisms in individual groups in the animal faeces (CFU/g).

Parameter	Experimental Group (n6)				SEM	*p*-Value
	0	I	II	III		
Total number of mesophilic bacteria	6.0 × 10^6^	1.6 × 10^7^	1.9 × 10^7^	1.1 × 10^7^	1.4 × 10^6^	0.075
Total number of fungi	1.2 × 10^3^	4.2 × 10^3^	1.8 × 10^3^	8.2 × 10^2^	8.6 × 10^2^	0.192
Total number of coliforms	3.5 × 10^6^	9.3 × 10^6^	4.3 × 10^6^	4.5 × 10^6^	7.8 × 10^5^	0.475
Total number of *E. coli*	2.7 × 10^6^	6.1 × 10^6^	2.8 × 10^6^	3.8 × 10^6^	2.6 × 10^5^	0.434
Total number of lactic acid bacteria of the genus *Lactobacillus*	2.4 × 10^6^	2.1 × 10^6^	2.1 × 10^7^	2.0 × 10^6^	3.1 × 10^5^	0.979
Total number of *C.* *perfringens*	1.2 × 10^4^	4.7 × 10^2^	5.8 × 10^2^	1.1 × 10^2^	1.2 × 10^3^	0.125

SEM–Standard error of the mean. Groups: 0—control; I—group receiving a diet with 2% FRSM; II—group receiving a diet with 4% FRSM; III—group receiving a diet with 6% FRSM.

## Data Availability

All relevant data are within the paper.
